# Sensitive Parameter Analysis for Solar Irradiance Short-Term Forecasting: Application to LoRa-Based Monitoring Technology

**DOI:** 10.3390/s22041499

**Published:** 2022-02-15

**Authors:** María C. Bueso, José Miguel Paredes-Parra, Antonio Mateo-Aroca, Angel Molina-García

**Affiliations:** 1 Department of Applied Mathematics and Statistics, Universidad Politécnica de Cartagena, 30202 Cartagena, Spain; mcarmen.bueso@upct.es; 2 Technologic Center of Energy and Environment, 30202 Cartagena, Spain; jmparedes@cetenma.es; 3 Department of Automatic, Electrical Engineering and Electronic Technology, Universidad Politécnica de Cartagena, 30202 Cartagena, Spain; antonio.mateo@upct.es

**Keywords:** LoRa technology, PV monitoring, sensitive parameter analysis

## Abstract

Due to the relevant penetration of solar PV power plants, an accurate power generation forecasting of these installations is crucial to provide both reliability and stability of current grids. At the same time, PV monitoring requirements are more and more demanded by different agents to provide reliable information regarding performances, efficiencies, and possible predictive maintenance tasks. Under this framework, this paper proposes a methodology to evaluate different LoRa-based PV monitoring architectures and node layouts in terms of short-term solar power generation forecasting. A random forest model is proposed as forecasting method, simplifying the forecasting problem especially when the time series exhibits heteroscedasticity, nonstationarity, and multiple seasonal cycles. This approach provides a sensitive analysis of LoRa parameters in terms of node layout, loss of data, spreading factor and short time intervals to evaluate their influence on PV forecasting accuracy. A case example located in the southeast of Spain is included in the paper to evaluate the proposed analysis. This methodology is applicable to other locations, as well as different LoRa configurations, parameters, and networks structures; providing detailed analysis regarding PV monitoring performances and short-term PV generation forecasting discrepancies.

## 1. Introduction

The high integration of variable renewable energy sources (vRES) into current power systems, mainly wind and solar photovoltaic (PV) power plants, can be a key component of the resulting low-carbon power systems. However, their intermittency requires more flexibility from the rest of the power system to maintain certain grid stability and reliability levels [1]. Consequently, the increase in demand variability created by such intermittent sources presents new challenges to provide relevant system flexibility [2]. In addition to these challenges, accurate forecasting of renewable generation is also required by both transmission and distribution system operators in order to mitigate the negative impact on the grids of such variable and noncontrollable resources. According to Daliento et al., similar models are usually adopted for power forecasting for PV fields, which is important for both monitoring purposes and the management of the utility grid [3]. With this aim, different solar PV power generation forecasting solutions can be found in the specific literature [4]. The forecasting timeframe, also called horizon, is firstly defined according to the grid operation and under both spatial and temporal resolutions. Different forecast horizons can be then defined, varying from seconds to days or weeks ahead. Regarding spatial horizons, they can also be spanned from single site to regional forecasts [5]. In [6], a new model based on hourly measurements is proposed and evaluated. In [7], the application of neural networks for photovoltaic power generation forecasting purposes is explored by Oudjana et al. Other forecasting neural network-based solutions can be found in the specific literature [8,9,10,11,12]. Naveed Akhter et al. [13] present a critical and systematic review of photovoltaic (PV) power forecasting methods, mostly focused on machine learning and metaheuristic based-solutions. An extreme learning machine technique is used for PV power forecasting of a real time model [14]. A revision of solar irradiance and PV power forecasting, both topics combined as “solar forecasting”, using text mining is discussed in [15]. Barbieri et al. [16] conclude that cell/module temperature and irradiance can be considered as the best approaches for an accurate PV power forecasting; mainly under cloudy conditions with hardly predictable power generation fluctuations. A probabilistic forecast review focused on inherently erroneous of different forecasting strategies is discussed and quantified in [17]. Short-term photovoltaic power generation forecasting is also an important task in renewable energy power system planning and operating. In fact, Kaur et al. [18] affirm that short-term electricity trading to balance demand and generation offers a remarkable economic opportunity to integrate larger shares of vRES into future power grids. A novel multi-timescale data-driven forecast model to improve the accuracy of short-term PV power production is proposed by Yang et al. [19]. Dambreville et al. [20] propose a new approach of global horizontal irradiance (GHI) forecasting for very short term by using a spatio–temporal autoregressive model.

From the specific literature, observed weather data are commonly applied on the solar PV generation forecasting model [21]. Subsequently, the solar PV forecasting model performance is then evaluated by quantifying discrepancies between such forecasts and the weather measurements through the use of traditional statistical error metrics, such as the mean bias error or the root mean square error (RMSE) [22]. For example, the mean absolute percentage error (MAPE), and mean absolute error (MAE) indicators are used in [23] to evaluate the performance of day-ahead photovoltaic power forecasting models based on deep learning neural network. Normalized root mean square error (nRMSE) is used in [24] to evaluate the forecasting errors. In a similar way, Huang et al. describe a comparative study of solar PV power forecasting methods based on nRMSE [25]. An extensive comparison of simple forecasting methodologies with more sophisticated solutions over 32 photovoltaic (PV) plants of different sizes and technology over a whole year is carried out by Gigoni et al. [26]. However, there is a lack of contributions focused on evaluating possible forecasting errors and minor accurate results derived from the inherent communication network failures: packet losses, possible packet collisions, etc., as well as the short time period influence of such GHI forecasting accuracy. Indeed, possible communication failure can significantly affect both gathering data and forecasting results [27]. Subsequently, and by considering such missed contributions in the specific literature about this issue, the present paper analyzes the influence of the LoRa solution—identified by the literature as a promise and suitable technology—on the GHI short-term forecasting accuracy, by considering real communication architecture/layout and specific LoRa performance characteristics. In addition, current datasets available from satellite-based installations, ground-based installations, and solar PV power plants connected to the grid are also considered for evaluation. In this way, a case example located in the southeast of Spain is included in the paper to assess the suitability of the proposed methodology. The main contributions of this paper can be summarized as (i) a methodology to evaluate GHI short-term forecasting accuracy for different node layouts and LoRa parameters based on a random forest prediction model; (ii) a sensitive analysis of LoRa parameters and short-term intervals on the GHI forecasting values based on a variety of metrics; and (iii) a case study from 2019 GHI data (one-minute sample time), 400 km${}^{2}$ area, 289 potential nodes under consideration, and a total of 13,140 simulations. This methodology thus provides a preliminary extensive analysis of potential LoRa network characteristics and node layout in terms of data accuracy, packets, and GHI forecasting possibilities before the installation was completed.

The rest of the paper is structured as follows. Section 2 discusses PV power plant monitoring and the use of LoRa technology as a solution to be implemented in such installations. Section 3 describes the proposed methodology. A case example is presented in Section 4. Results and discussion are provided in Section 5. Finally, conclusions are given in Section 6.

## 2. Pv Monitoring: LoRa Communication Technology

### 2.1. General Overview

A variety of PV monitoring strategies based on the output PV power plants and their nature have been proposed in the literature; being performed remotely or locally on site. New advanced monitoring techniques are continuously under investigation; mainly due to the evolution and relevant integration of PV installations into power systems. A recent PV monitoring review is analyzed by Triki-Lahiani et al. [28]; discussing their differences, advantages, and limits. An impedance-based monitoring method for detection of distribution system current behavior is presented in [29]. This monitoring technique can be used for small variation of PV penetration level, and for some fast transient detection, such as the effect of cloud movement on a PV system.

### 2.2. Lora-Based Communication System

Although different wireless technologies—such as Bluetooth, Zigbee, Wi-Fi, GSM, Sigfox, or LoRa—have been evaluated for PV solar monitoring through wireless sensors networks, LoRa technology has been chosen as the wireless technology due to its long range and low power consumption [30]. Moreover, this technology has received significant attention in recent years from network operators and solution providers [31,32,33,34]. An impartial and fair overview regarding the capabilities and the limitations of LoRaWAN is discussed by Adelantado et al. [35] to clarify its comprehension and avoid inflated expectations. LoRa uses six spreading factors (SF07 to SF12) to adapt the data rate and range trade-off. It can be affirmed that a higher spreading factor (SF) allows longer range at the expense of lower data rate [36]. The LoRa data rate depends on the channel bandwidth and the SF, ranging from 0.3 kbps to 27 kbps [37]. According to Mikhaylov et al. [38], messages transmitted with different SFs can be received simultaneously by LoRa base stations, 243 bytes being the maximum payload length for each message.

A collision behavior model, C(x, y), for LoRa network between node *x* and node *y* is proposed by Bor et al. [39]. A collision rate analysis with a single LoRa gateway was reported by Alenezi et al. [40]—depending on the number of nodes and the SF for one (20 byte) packet each hour for 24 h (125 kHz bandwidth). In [41], Zhang et al. propose an alternative low-power wide area network information monitoring approach based on LoRa and NB-IoT. In this case, the communication distance in a complex environment was up to 1.6 km, with a system communication packet loss rate of around 3%. Silva et al. [42] test LoRa (long range) technology and LoRaWAN protocol in a precision viticulture scenario using low-power data acquisition devices, distanced 400 m away from the nearest gateway. With regard to the communication area parameter, Liu et al. [43] present a low-power, real-time air quality monitoring system also based on the LoRa technology being able to reach to approximately 2 km. A range of 9.27 km was achieved with SF12 and a bandwidth of 125 kHz in [44], for module-level monitoring of solar PV plants. The LoRa communication usefulness for monitoring the climate information of PV power plants was tested by Jeong et al. [45]. From the test results, it can be affirmed that the communication range for the PV climate information transmission reaches 1.3 km. In general, it can be assumed that LoRa radio chipsets use a maximum of 100 mW when transmitting, with a range of 10 to 30 km in suburban areas. In [46], it is affirmed that LoRa performs much better in comparison to FSK in most scenarios. It also highlights the successful transmission ranges within suburban scenarios up to 10 km.

## 3. Methodology

### 3.1. Lora Parameter Modeling

In the specific literature, a review of LoRa simulation environments can be found in [47], where aspects such as the selection of LoRa parameters or the device energy consumption were revised and compared. For example, LoRaSim was described as a well-known custom-built discrete-event simulator implemented with SimPy [39]. It allows us to place a group of *N* LoRa-nodes and *M* LoRa-stations in a bidimensional space. A directional antennae is also considered for simulation purposes and identification of LoRa parameters. In this way, a comparison between the use of directional antennae facing multiple base stations as methods of dealing with LoRa internetwork interference is carried out in [48]. However, these solutions do not analyze the influence and sensitive of LoRa nodes on the PV forecasting accuracy, under different communication errors/discrepancies and a diversity of LoRa node layouts.

The transmission parameters define the LoRa node communication characteristics. As discussed in Section 2.1, LoRa provides three bandwidth (BW) settings—125, 250, or 500 kHz, and six different spreading factor (SF) values. According to Croce et al. [49], a larger bandwidth translates to a data rate increase and a receiver sensitivity deterioration. Conversely, higher SFs can be used to improve the link robustness at the cost of lower data rates. LoRa modulation is derived from chirp spread spectrum (CSS). LoRa CSS modulations with BW of 125 kHz are assumed in this work, 1% duty cycle, and a default radiated transmit power of 14 dBm. Croce et al. [50] identified six different SFs: from SF07 to SF12. In Europe, both 868 MHz and 433 MHz bands are allowed to be used. Transmitted power is limited to 14 dBm effective isotropic radiated power (EIRP), with a 1% duty cycle limit of on-air time, and the transmitted power limited to 14 dBm effective isotropic radiated power (EIRP). Table 1 summarizes the LoRa/LoRaWAN main characteristics [51]. According to the specific literature, there is pseudo orthogonality among SFs, having the advantage that multiple signal reception is possible [52]. Table 2 shows the estimated range for different SFs (from SF07 to SF10) that can be used for uplink messages on a 125 kHz channel depending on the terrain: longer distances can be achieved in a rural environment than in an urban environment [53].

For a specific SF, the narrower the bandwidth, the higher the receiver sensitivity [54]. Consequently, the data rate selection is then considered as a trade-off between message duration and communication range. Different tools allow us to estimate time on air and optimum bandwidth. For the present proposal, the time interval on air for a 51-byte payload for each specific SF is considered. The payload size is defined as the maximum payload length. For each transmission, the payload can range from 2 to 255 octets, reaching the data rate up to 50 kbps when channel aggregation is used [55] under the assumption that any packet arrivals follow a Poisson law—thus, considering a uniform distribution of the payloads, their lengths are between 1 and 51 bytes [56]. According to Centenaro et al. [57], it can be assumed that the data transmission in a LoRaWAN presents a typical 1% duty-cycle constraint; from the nodes to their corresponding gateways in a single hop allocated on different sub-bands. Indeed, the European regulations currently ask for adherence to 1% duty cycle per sub-band or applying any mechanism based on “listen-before-talk and adaptive frequency agility” [58]. Table 3 summarizes the corresponding time intervals between subsequent starting packets (s) for a 1% duty-cycle.

As previously described, LoRaWAN is built as a star-of-stars topology, where the devices located in the defined grid are able to send packets to a gateway which is then responsible for forwarding those packages to a network server [59]. It is assumed that each end-device selects a specific SF based on the data rate and the distance to the gateway. A radial equidistant distribution with homogeneous end-device density is thus considered, being the energy consumption of *j*-radial annulus proportional to the airtime. In order to give a more realistic simulation, the study of LPWAN modeling proposed by Georgiou and Raza [60] is used to analyze the capability of this technology to scale. This study also includes an outage probability model which occurs at the gateway, called outage condition [61]. Table 4 gives the outage of a desired signal in the uplink that can occur at the gateway, if the received signal to noise ratio (SNR) is below the SF specific threshold.

It can be then determined the packet delivery ratio, defined as the ratio between the client of packages originated by the “application layer” and the number of packages received by the sink at the final destination [62]. From this parameter, we obtain the probability function that any packet should be lost. We consider a Rayleigh channel, in line with Duda and Heusse [63]. The received signal power is affected by a multiplicative random variable with an exponential distribution of unit mean (and standard deviation). Consequently, the signal power depends on the Rayleigh fading gain and the distance, keeping the noise power constant for a 125 kHz wide band (N=−123 dBm). A maximum transmission power of P=14 dBm is considered for simulations; the successful transmission probability being with data rate $DR_{j}$ and at distance $l_{j}$:

(1) $$H\left(l_{j}\right)=exp\left(\frac{N\cdot q_{j}}{P\cdot g(l_{j})}\right),$$

where $g(l_{j})$ is the average channel gain at distance $l_{j}$, *P* is the transmission power (in dBm), $q_{j}$ is the signal to noise ratio (SNR) threshold for $DR_{j}$, and *N* is the wide band (in dBm). The path loss attenuation is estimated by using the Radio Mobile software package [64]. It uses the terrain information and the mathematical model to calculate the coverage area from the fixed radiation point taken as mobile reference point [65]. The irregular terrain model (ITM) is used as a propagation model. It estimates radio propagation losses over irregular terrain in the range 0.020 to 20 GHz frequencies as a function of space and distance and the variability of signal in time [66]. The Okumura–Hata model [67] has also been recently proposed and assessed for path loss attenuation, mostly focused on comparing the performance LoRaWAN analysis in urban scenarios.

In the simulations, it is assumed that all transmitters (i) send packets with the same payload length; (ii) do not switch the SF from one packet to another during the same simulation test—despite that the adaptive data rate is one of the main strengths of LoRa [68]; (iii) do not change the transmit power from one packet to another during the same simulation test; and (iv) all of the transmitters have the same number of packets to send. An example of maximum communication ranges on ground (15 km) and on water (30 km) can be found in [69], including packet loss ratio, depending on the distance and assuming maximum signal SF—868 MHz ISM band using 14 dBm transmit power.

### 3.2. Spatio–Temporal PV Forecasting

As discussed in Section 1, different probabilistic models for spatio–temporal PV forecasting can be found in the specific literature. However, only a few spatio–temporal models for short-term probabilistic forecasting can be identified [70], which are based on regression trees [71], the vectorial autoregressive model and gradient boosting combination [72], the kNN method [73], multivariate predictive distributions [74], and Gaussian random fields [75]. A least absolute shrinkage and selection operator (LASSO) regression method was also presented by Yang et al. [76] for sub–5–min solar irradiance forecasting. A flexible spatio–temporal model to estimate PV production forecasts was recently proposed by Agoua et al. [77] for horizons up to 6 h ahead, evaluating the effect of different spatial and temporal data sources on the accuracy of the forecasts. According to Muhammad et al. [78], the ARX model is the simplest black box linear model, based on a structure that is known as the most common input–output model. In this work, the author selected the random forest (RF) approach, mainly due to its simplicity to deploy, low computational cost, and ease in interpreting the input interactions. The RF algorithm is then used to find an adequate predictor function *f*. The RF algorithm is an ensemble learner, proposing a set of decision trees that vote on a final result. Dudek [79] affirms that this model operates on patterns of the time series seasonal cycles, considerably simplifying the forecasting problem—mainly when a time series exhibits heteroscedasticity, trend, nonstationarity, and multiple seasonal cycles. To train and test the RF algorithm, different partitions of the data are used accordingly. Firstly, the parameter-tuning process of the RF learning is carried out through the training set. Subsequently, the test set is used to estimated the final metrics. An R package ranger to be used as a fast RF implementation for high-dimensional data is used in this work [80].

**Figure 1:**
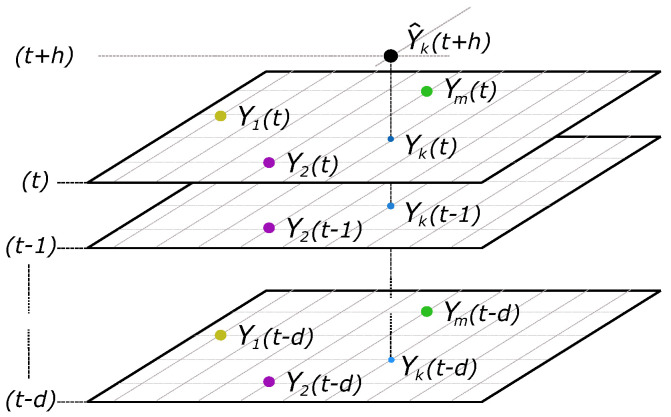
Smart persistence and RF model proposed for spatio–temporal forecasting.

**Figure 2:**
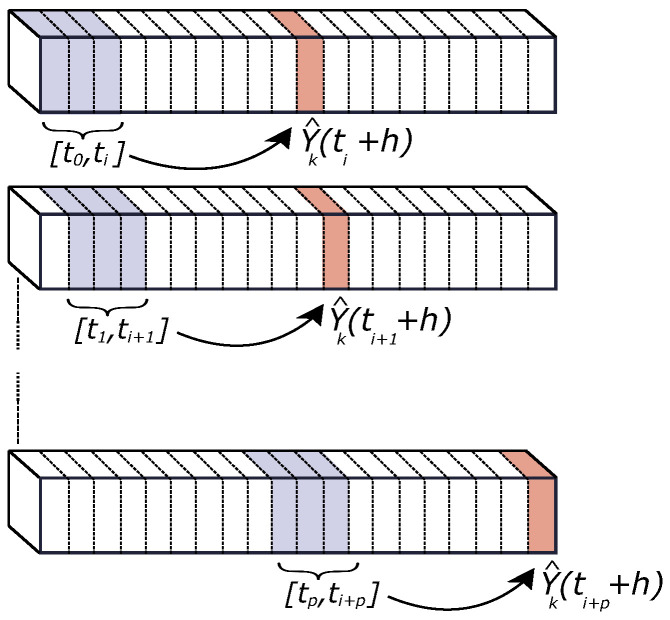
Forecast training strategy for a prediction horizon (*h*).

**Figure 3:**
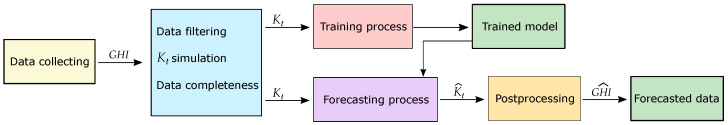
Training and forecast processes from collected data by using the clear sky index ($K_{t}$).

By considering the aim of this paper, a smart persistence and RF model is proposed for spatio–temporal PV forecasting, from the general expression given for time series [81],

(2) $${\hat{Y}}_{k}\left(t+h\right)=f\left(Y_{k}\left(t\right),Y_{k}\left(t-1\right),Y_{k}\left(t-2\right),\ldots ,Y_{k}\left(t-d\right)\right),$$

where ${\hat{Y}}_{k}\left(t+h\right)$ is the $k^{th}$ sensor forecasting and time step t+h, *h* is the horizon (in minutes) for which the prediction is being made, $Y_{k}\left(t-l\right)$ is the data past collected at the $l^{th}$ lag, l=0,…,d. This general expression can be generalized and further extended to include other relevant information about radiation, weather, statistical measures, etc.

(3) $$\begin{array}{c} {\hat{Y}}_{k}\left(t+h\right)=f(Y_{1}\left(t\right),Y_{1}\left(t-1\right),Y_{1}\left(t-2\right),\ldots ,Y_{1}\left(t-d\right),\\ Y_{2}\left(t\right),Y_{2}\left(t-1\right),Y_{2}\left(t-2\right),\ldots ,Y_{2}\left(t-d\right),\\ \ldots ,\\ Y_{m}\left(t\right),Y_{m}\left(t-1\right),Y_{m}\left(t-2\right),\ldots ,Y_{m}\left(t-d\right)), \end{array}$$

being $Y_{j}$, for j=1,…,m, the predictors to be used and $Y_{j}\left(t-l\right)$ a single predictor (see Figure 1), and *h* the prediction horizon. This extended expression allows us to use additional information aside from the PV production data. Different measurement scenarios are then proposed and evaluated for GHI forecasting comparison purposes, as described in Section 3.3. GHI estimated data are thus forecast on short time horizons from 15 to 45 min. The time steps are set as 1 min, and *d* = 15 min. It means that, to forecast ${\hat{Y}}_{k}\left(t+h\right)$, as can be seen in Figure 1, the time interval data from *t* to t−15 is considered as input for prediction. The *d* parameter can be modified depending on the time step and the number of nodes (from 1 to *m*) included in each case study. The parameters are estimated according to the prediction training strategy depicted in Figure 2. The clear sky index $K_{t}\left(t\right)$ is used in the forecasting model. Assuming to be stationary enough, it is defined as follows:

(4) $$K_{t}\left(t\right)=\frac{GHI(t)}{GHI_{sc}\left(t\right)},$$

being, thus, the ratio of the measured GHI to GHI under clear sky conditions (GHI${}_{sc}$). The training and forecasting processes are summarized in Figure 3, in line with recent studies also focused on machine learning forecast model analysis applied to solar power forecasting [82].

### 3.3. Proposed Global Methodology

**Figure 4:**
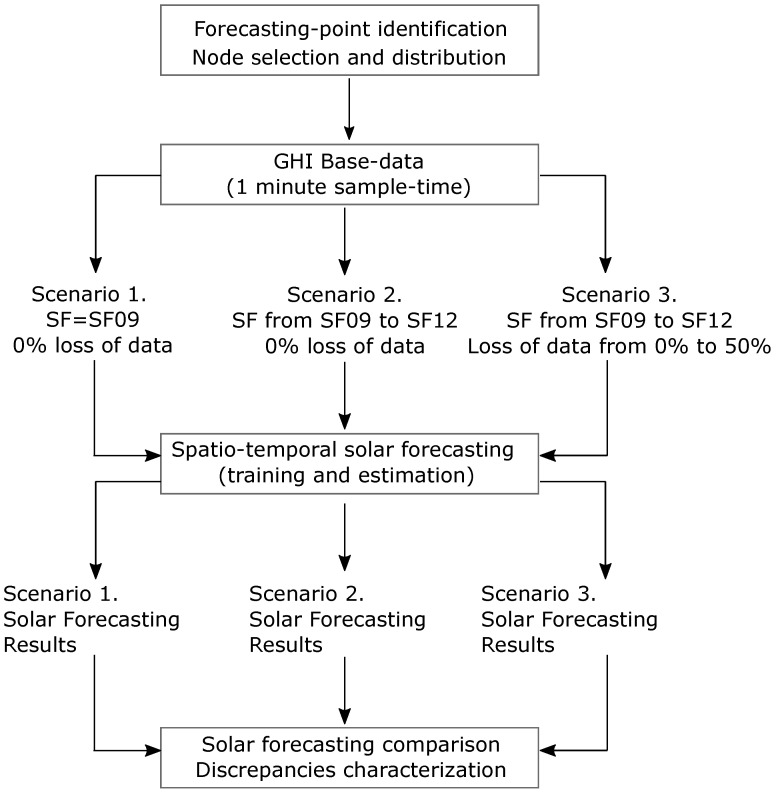
Proposed methodology: general scheme.

This work aims to evaluate the influence of LoRa performance characteristics and its architecture/layout on the short-term PV forecasting, by considering the RF model described in Section 3.2. Firstly, a node selection and distribution based on LoRaWAN technology is carried out according to a predefined forecasting point of interest and a possible group of potential on-ground sites or satellite based-installations. From these specifications, a one-minute sample time database is defined on each node, as well as the forecasting point of interest. The RF algorithm is used to find a suitable predictor function *f* and, then, to forecast short-term solar values on such location by considering different forecasting time intervals—from 15 to 45 min. These predictions are estimated under a variety of scenarios: (i) assuming SF = SF09 for all nodes and 0% loss of data; (ii) assuming SF from SF09 to SF12 on each node and 0% loss of data; and (iii) assuming SF from SF09 to SF12 on each node and loss of data from 0% to 50%. Subsequently, the solar forecasting values corresponding to the different scenarios are then compared. Discrepancies and similarities are calculated, discussing the influence of the different realistic LoRa parameters on the solar forecasting process. As previously analyzed by the authors in [83], different metrics can be found in the specific literature to determine discrepancies. From this classification, normalized root mean square error (nRMSE), mean absolute percentage error (MAPE), and dynamic time warping (DTW) are determined to provide complementary information and characterize convenient discrepancies among the PV short-term forecasting data by considering SF09 and 0% loss of data and the rest of scenarios. Figure 4 summarizes the proposed methodology. In addition, a sensitive analysis based on SF parameter and loss of data variability is also included, determining the differences among discrepancies with respect to the forecasting PV values with SF09 and 0% loss of data. The methodology and simulations were implemented in the R–environment [84]. Different contribution software packages were used for methodology implementation purposes. In this case, data.table for fast and memory efficient data manipulation [85], ranger for a fast RF implementation [80], and dtw and dtwclust for the DTW metric estimation [86].

## 4. Case Example: Datasets Used

**Figure 5:**
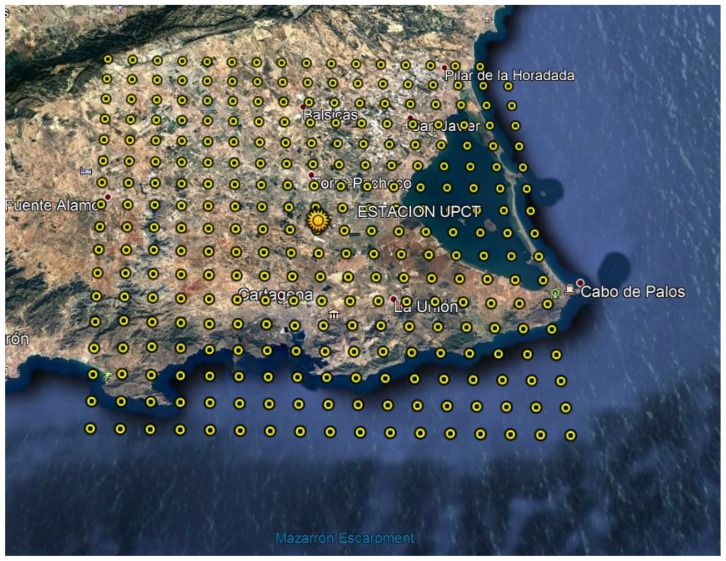
Case example: general overview and datasets (Region of Murcia, Spain).

**Figure 6:**
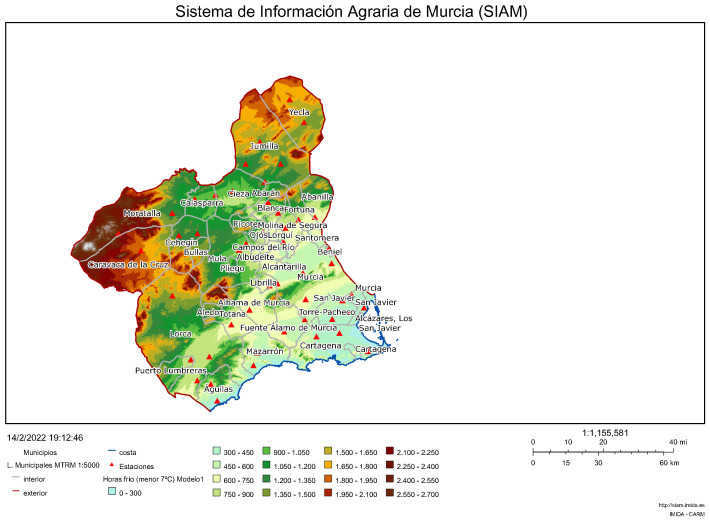
Case example: ground-based data available online (Region of Murcia, Spain) [90].

Nonnenmacher et al. [87] affirm that satellite images based on prediction methods are mostly used for intraday forecasts lower than four hours. Based on this assumption, a total area of 400 km${}^{2}$ located in the Region of Murcia (11,300 km${}^{2}$, southeast of Spain) is selected as a case example. This region is a promising area to integrate solar PV power plants, with 5.2 kWh/m${}^{2}\cdot$day as averaged annual global irradiation [88]. Figure 5 shows a general overview of this area, covering a 17 × 17 grid portion with a total of 289 points under consideration and a forecasting point identified in the center of this grid that is selected for GHI estimated data—corresponding to ${\hat{Y}}_{k}\left(t+h\right)$ according to expression (2). The considered spacing between all pairs of grid points is assumed as 2.5 km. Day-ahead GHI estimated data are downloaded from the Copernicus European Project servers [89]—from January to December 2019. In addition, ground data are also available based on the Network of the Agricultural Information System of the Region of Murcia (SIAM), giving additional ground-based GHI data. The SIAM network consists of 49 ground-based automatic stations geographically distributed; 32 stations are from the Regional Murcia Institute of Agricultural and Food Research and Development (IMIDA), 15 stations are from the Spanish Ministry of Agriculture, Food and Environment, one station is from the Universidad Politécnica de Cartagena (Murcia, Spain), and one more is from the City Council of Mazarrón (Murcia, Spain). These ground-based stations are financially supported by several European fund projects [90], see Figure 6. A ground-based station located in the center of the grid corresponds to the forecasting point considered for this GHI forecasting analysis.

**Figure 7:**
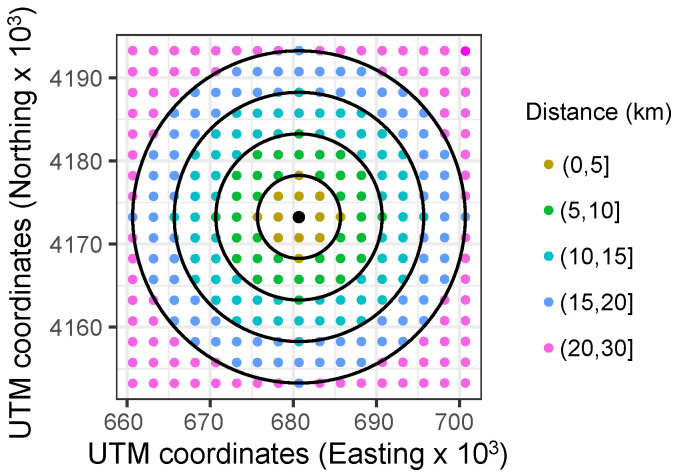
Case example: distribution of datasets (UTM coordinates).

According to the proposed methodology described in Section 3.3, the LoRa node distribution is then selected by considering the initial grid depicted in Figure 5. With this aim, Figure 7 gives a general overview of the distribution of potential nodes, including UTM coordinates. Duda and Heusse [63] affirm that a more realistic assumption is to consider that the node density decreases with the inverse square of the gateway distance: a specific intensity of physical quantity is inversely proportional to the distance square from the source. Nevertheless, one node by each circular crown is considered and, thus, a homogeneous and minimum density distribution is considered to be evaluated and compared for short-term PV forecasting purposes. With regard to the LoRa parameters, and as can be found in the specific literature, the lowest transfer rate ensures the highest level of collisions [91]. Indeed, this configuration is the most used one, as it ensures the largest communication range by using a high SF (e.g., SF = 12). Therefore, a trade-off is then determined between increasing the communication range and reducing the transfer rate. As described in Section 3.1, different SF values are also considered in the different conditions. Therefore, different scenarios are considered for each day from the initial grid depicted in Figure 7, with forecast horizons ranging from 15 to 45 min with one-minute time resolution, and under a variety of SF LoRa parameters and loss of data values (see Figure 4). A general comparison of GHI prediction results for the case study is following discussed in Section 5.

## 5. Results

**Figure 8:**
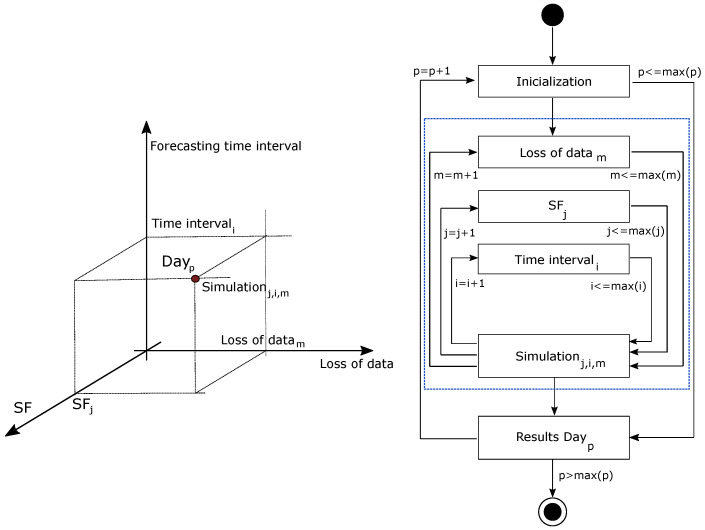
Case example: simulation general scheme.

**Figure 9:**
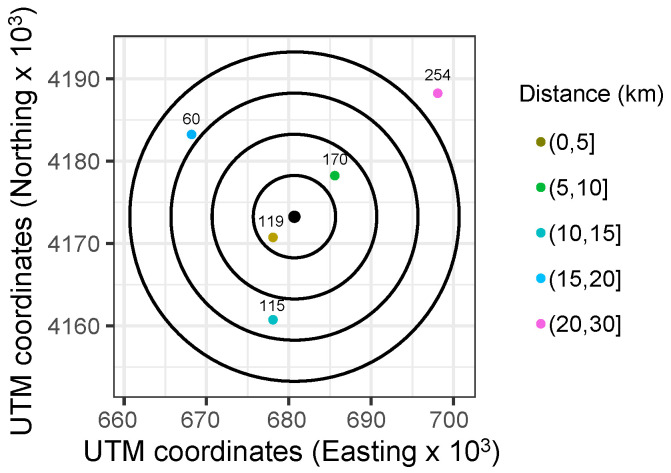
Case example: distribution of points selected and the forecasting point marked in black color (UTM coordinates).

From the data corresponding to 2019, with one-minute time resolution, and as described in Section 4, different short-term PV solar forecasting periods were considered for simulations. More specifically, three different time horizons were defined: 15, 30, and 45 min. Each day was then simulated by considering such different time horizons for forecasting purposes. In addition, and with the aim to compare the impact of both loss of data and the selected SF, simulations were carried out under such conditions: 0%, 25%, and 50% loss of data; as well as from SF12 to SF09. Indeed, and according to the discussion given in Section 3.1—see Table 2 and Table 3, the selected SF considerably affects the robustness at the cost of lower data rates. These results allow us to evaluate the impact of each parameter and give a preliminary analysis of the influence of these conditions and situations before implementing a real communication and sensoring network. Therefore, each day is simulated through a 3 × 4 × 3 matrix of possible loss of data, foresting time intervals and selected SF, as schematically depicted in Figure 8. Subsequently, 36 different conditions are considered for each day, including three different losses of data percentages, four different SF parameters, and three different forecasting time intervals. In line with the case example shown in Figure 5 and the initial node layout based on UTM coordinates and depicted in Figure 7, an arbitrary node site location is selected and given in Figure 9, including the distribution of selected points for the analysis and the distance to the forecasting point. Subsequently, and as previously discussed, one node is selected on each circular crown with the aim of forecasting GHI data in the center of the grid, corresponding to the $\hat{Y}\left(t+h\right)$—see expression (3). The selected nodes are labeled as 119, 170, 115, 60, and 254, respectively.

**Figure 10:**
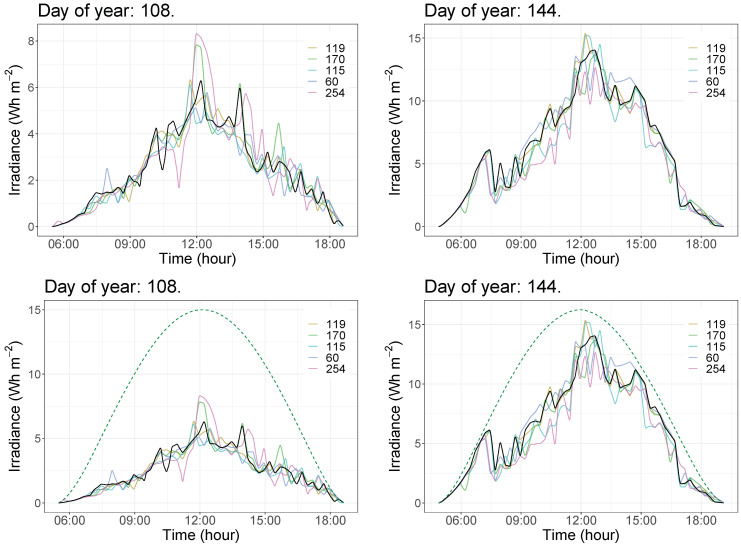
Example of irradiance data for two days: curves used for short-term forecasting purposes—clear sky GHI data included in dashed line. Curve in the forecasting point is marked in black color.

**Figure 11:**
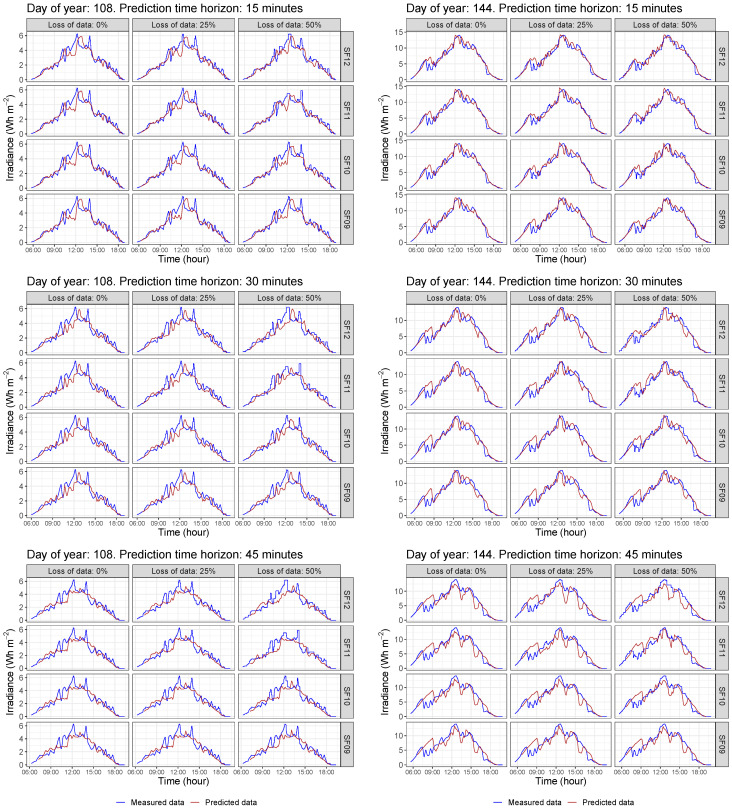
Comparison of estimated and monitored GHI data for different time horizon, SF, and loss of data scenarios.

**Figure 12:**
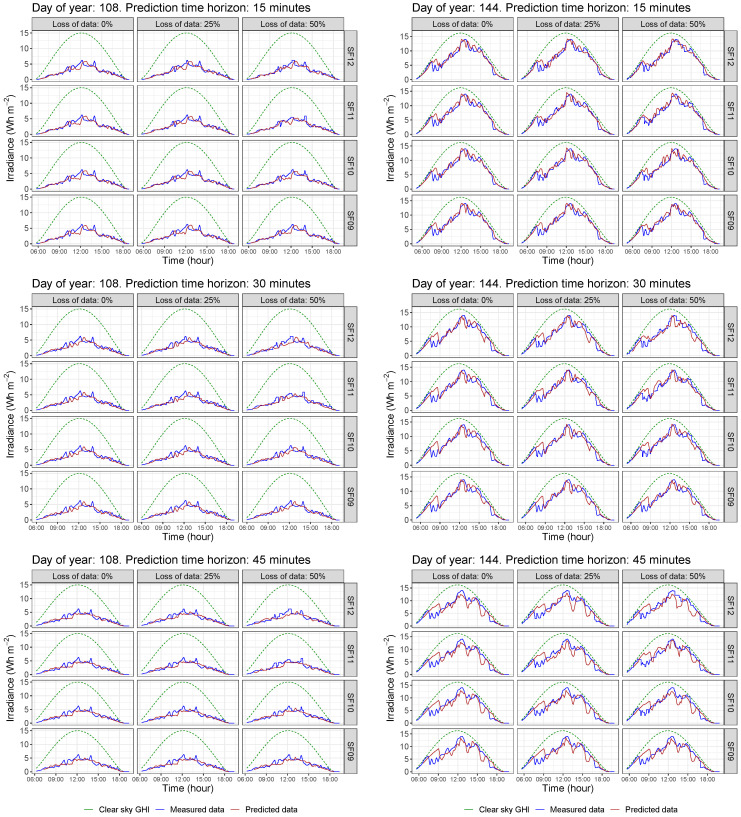
Comparison of estimated, monitored, and clear sky GHI data for different time horizon, SF, and loss of data scenarios.

By considering the 2019 data for the methodology evaluation, the corresponding daily GHI curves are then forecast according to the preliminary selection of possible nodes and the different conditions. In summary, a global of 13,140 simulations were carried out by the authors. These estimated data allow us to analyze in detail the influence of each variable on the short-term forecasting accuracy and the different possibilities to implement a real sensoring network in terms of data gathering accuracy and reliability for forecasting purposes. As an example of the forecast curves for each day, Figure 10 shows the irradiance data corresponding to two arbitrary days—labeled as day 108 and 144, respectively, including clear sky GHI data—see dashed line. As can be seen, data corresponding to the selected nodes given in Figure 9 are plotted, as well as the forecasting point marked in black color being the GHI observed data—corresponding to $Y_{k}\left(t+h\right)$. From these initial data, Figure 11 compares the measured and forecast irradiance values for the two previous days—labeled as day 108 and 144—and considers the selected different conditions for each day: loss of data percentages, different SF parameters, and different forecasting time intervals. Subsequently, 36 different forecasting GHI results are determined for each day. In addition, as a complementary result, Figure 12 compares these curves, including the expected clear sky GHI values. These forecasting data are thus determined for 15, 30, and 45 min time horizons, varying the SF parameter from 09 to 12 and considering 0%, 25%, and 50% loss of data scenarios.

**Figure 13:**
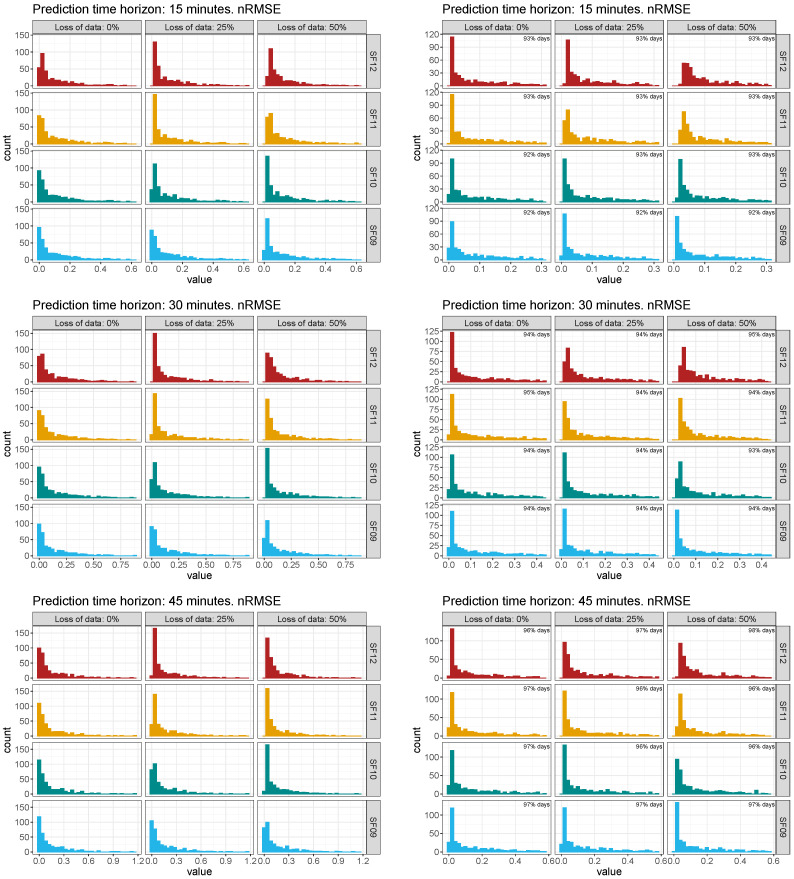
Summary of discrepancies for forecasting and monitoring data based on nRMSE. Histogram (**left**) and truncated histogram (**right**).

**Figure 14:**
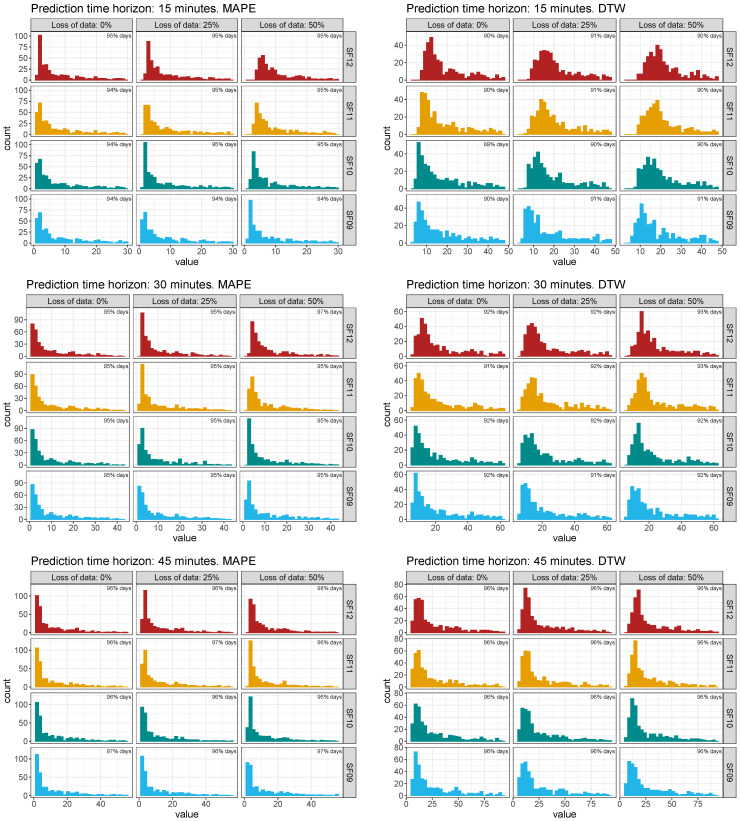
Summary of discrepancies for forecasting and monitoring data based on MAPE and DTW. Truncated histograms.

With the aim of estimating the influence of each parameters by considering all simulations along the 2019 data, a sensitive analysis was carried out determining discrepancies between the estimated daily GHI values for the selected node and the corresponding daily measured GHI values. Firstly, Figure 13 shows the global histograms and the truncated histograms of such discrepancies based on normalized root mean square error (nRMSE) among the measured GHI data and the forecasting GHI values for the selected node and by considering the different time horizon, SF, and loss of data scenarios. Therefore, 36 global and truncated histograms were determined from the 13,140 simulations. As can be seen, the truncated histograms retain more than 90% of such discrepancies and are considered suitable enough for this sensitive analysis. Secondly, and according to the variety of errors and differences available in the specific literature, as well as the comparison conducted by the authors in [83], the mean absolute percentage error (MAPE) and dynamic time warping (DTW) were also selected as metric estimations—see Section 3.3. Indeed, and as can be found in [92], DTW is considered as an appropriate technique to estimate and find an optimal alignment between two time-dependent sequences under a set of restrictions. DTW was initially used to compare different speech patterns, and also successfully applied in other fields, such as information retrieval and data mining. Additionally, DTW can be applied to detect and cope with different speeds and time deformations associated with time-dependent data. Recently, the R package IncDTW based on the DTW improved the possibilities to classify time series or clusters [93]. To analyze in detail the influence of each variable, Figure 14 shows the truncated histogram for these simulations using discrepancies based on MAPE and DTW metrics. As can be seen from these discrepancies, a higher loss of data range involves more relevant discrepancies, and these values present a secondary peak from 20% to 30% values. In terms of SF parameter, the vast values of discrepancies slightly shift from the [0, 10] interval (for SF09) to [10, 20] interval (for SF12). Therefore, a longer time interval between subsequent packets—see Table 3—implies higher discrepancy errors. These results are similar for the other analyzed forecasting short time intervals—30 and 45 min, respectively, as summarized in Figure 14.

**Figure 15:**
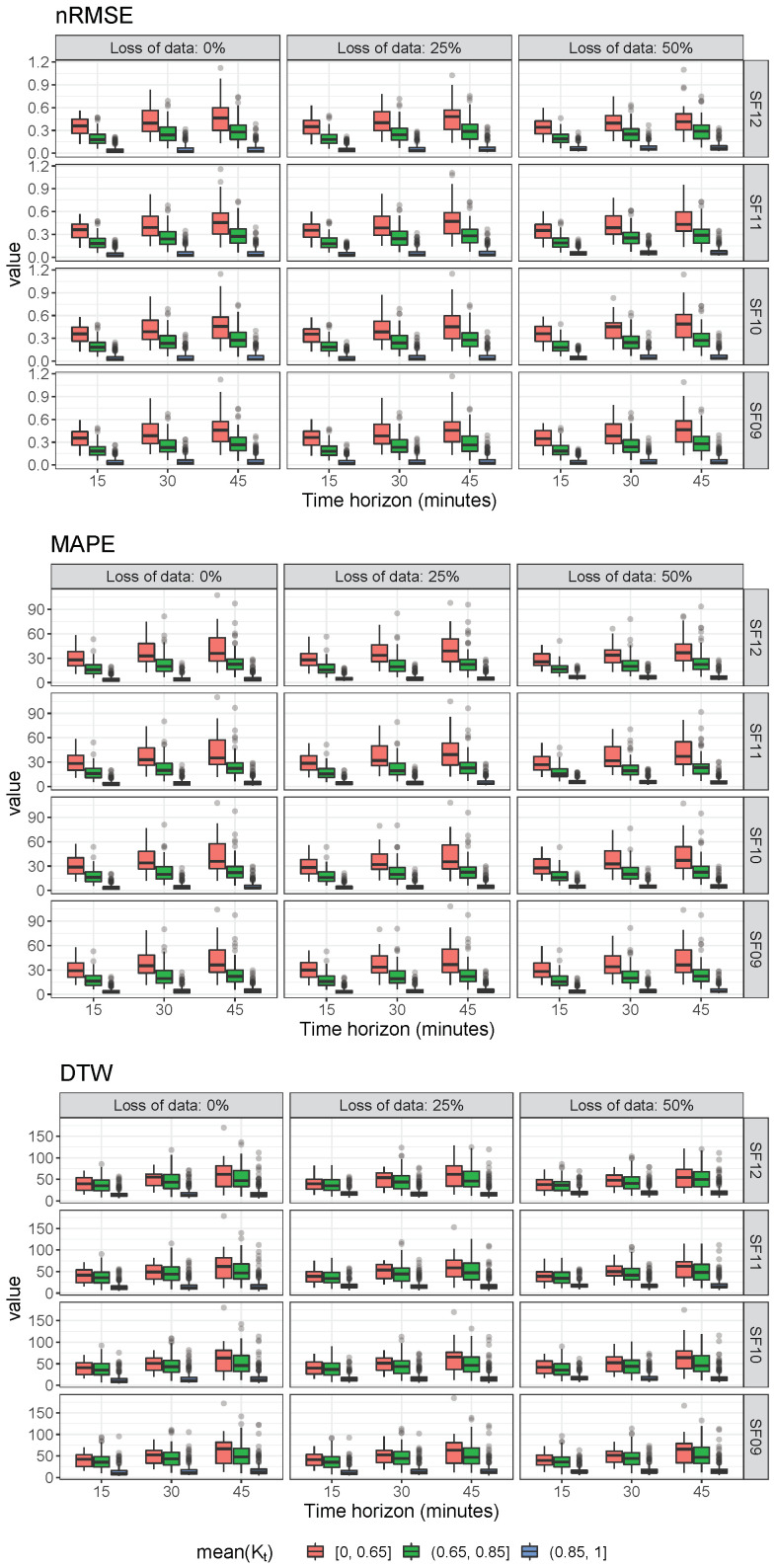
Boxplot of the discrepancies based on nRMSE, MAPE, and DTW.

As an alternative sensitive analysis, Figure 15 shows a boxplot diagram of the discrepancies based on nRMSE, MAPE, and DTW metrics classified according to different clear sky GHI ratio ${\bar{K}}_{t}$: [0, 0.65], (0.65, 0.85], and (0.85, 1], respectively. All simulations corresponding to 2019 data are considered to determine these global metrics; thus, 36 different conditions are assumed for each day depending on the defined 3 × 4 × 3 matrix of possible loss of data, SF parameter, and short time period values selected for this case study. From the results, larger time horizons address less-accurate GHI estimations, and higher clear sky GHI ratio values imply more accurate GHI estimations. Moreover, high ${\bar{K}}_{t}$ GHI ratio days should be not considered for LoRa network performance evaluation, as they are not sensitive to loss of data nor SF parameter values. Subsequently, low ${\bar{K}}_{t}$ GHI ratio days should be considered for estimating discrepancies and GHI forecasting accuracy, as well as potential errors allowed for short-term forecasting purposes depending on the corresponding loss of data, short time periods, and SF parameters.

**Figure 16:**
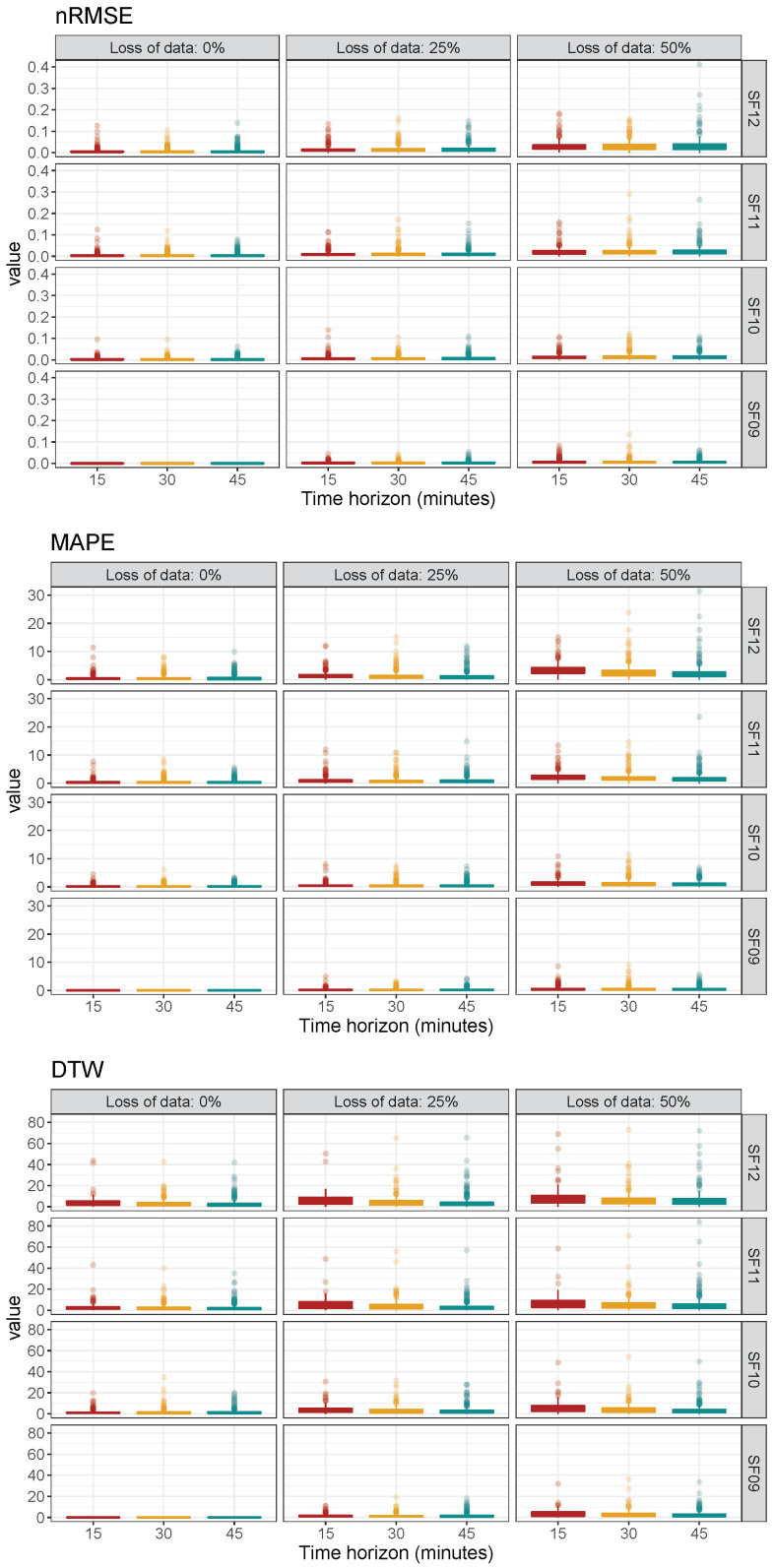
Boxplot of differences based on SF09 and 0% loss of data: nRMSE, MAPE, and DTW metrics.

**Figure 17:**
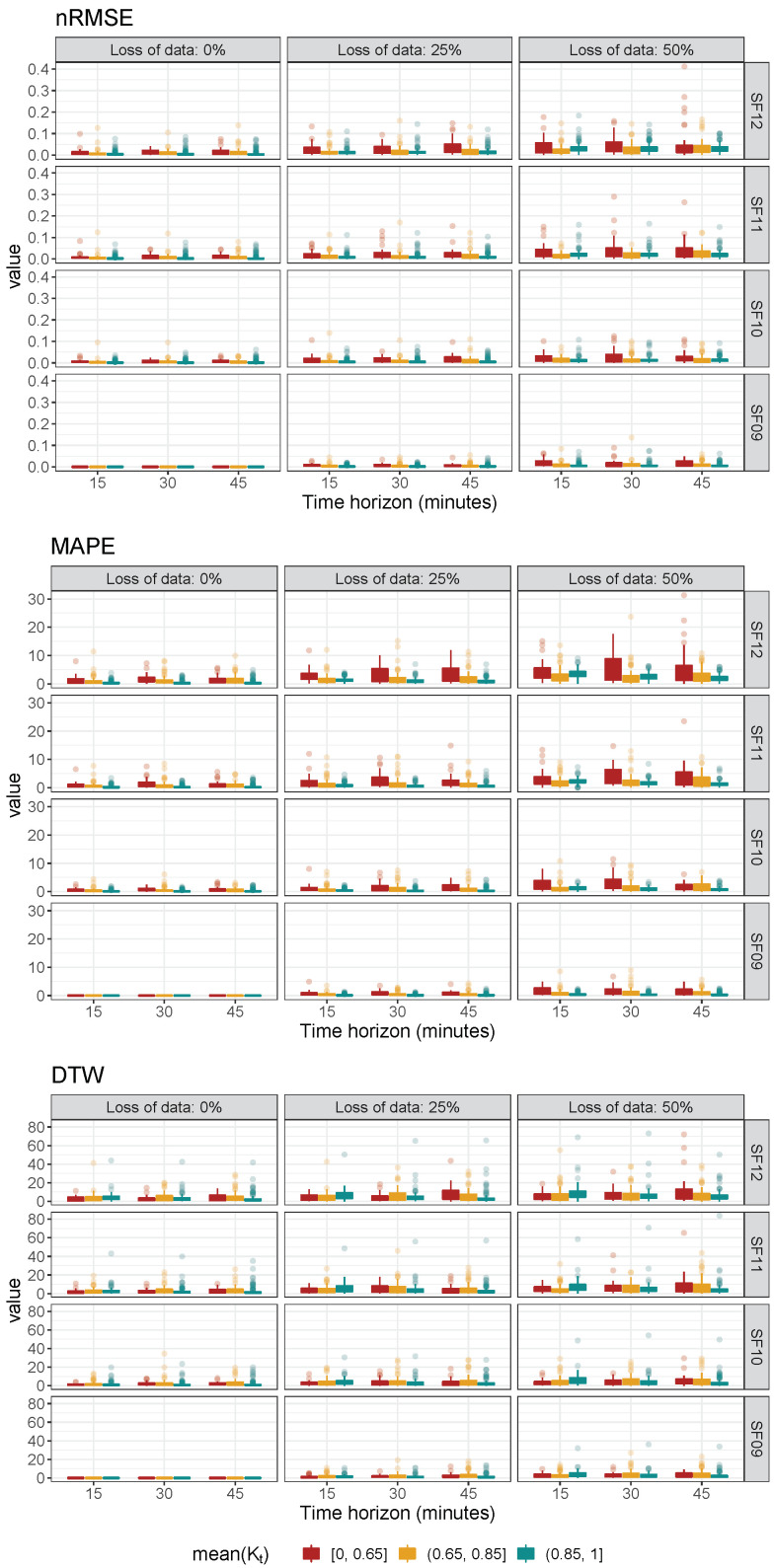
Boxplot of differences based on SF09 and 0% loss of data: nRMSE, MAPE, and DTW metrics for different $K_{t}$ clear sky GHI ratios.

Finally, an additional metric analysis is also provided to compare the difference evolution of SF09, 0% loss of data (corresponding to “Scenario 1” in Figure 4), and for each short time interval to the rest of the other daily possible conditions and under nRMSE, MAPE, and DTW metric estimations. With this aim, Figure 16 summarizes the boxplot results of differences among “Scenario 1” (for each short time interval) and the other daily conditions (SF values, loss of data, and short-term time intervals). As can be seen, differences are higher with larger loss of data for any metric—see boxplots for the same row and short time interval. In addition, discrepancies are increasing from SF09 to SF12, when loss of data and short time interval variables are kept constant. The results under different daily conditions are divided by considering clear sky GHI ratio intervals ${\bar{K}}_{t}$—[0, 0.65], (0.65, 0.85], and (0.85, 1]. The corresponding boxplot differences for SF09, 0% loss of data, and for each short time interval in comparison to the rest of daily conditions and for all metrics are determined and depicted in Figure 17. In general, differences are higher for lower ${\bar{K}}_{t}$ values and, as previously affirmed, low ${\bar{K}}_{t}$ GHI ratio days should be considered for evaluating GHI forecasting accuracy approaches and suitable node layouts. Consequently, and depending on the discrepancies range allowed in each case by the specific application, this methodology gives a preliminary analysis for the LoRa-based PV monitoring architectures and potential node layouts. Additionally, it gives an estimation of forecasting GHI estimations and the influence of SF and loss of data variables on the GHI value accuracy. Therefore, both reliability and robustness of the collected data to be used for forecasting purposes are able to be analyzed and evaluated.

## 6. Conclusions

A methodology to evaluate different LoRa-based PV monitoring configurations in terms of short-term GHI forecasting characterization of metrics is described and assessed. A location analysis of nodes based on potential loss of data, short-term time intervals, and SF ranges are considered to analyze their influence on such short-term GHI forecasting purposes. The methodology also allows us to evaluate different node layouts and forecasting approaches. A random forest model is proposed in this work as suitable forecasting method under nonstationary and multiple seasonal cycle data. A case study located in the southeast of Spain is included to evaluate the methodology. Satellite-based GHI data collected for 2019 covering a 17 × 17 grid portion with a total of 289 points under consideration are considered, with one-minute sample time data. The short-term GHI forecasting simulations for each day included different loss of data ranges, forecasting time intervals (15, 30, and 45 min), and SF values (from SF09 to SF12). A total of 36 different conditions for each were considered, running 13,140 simulations for the corresponding global 2019 GHI data. The results allow us to explore the influence of loss of data, SF values, and short-term time intervals on the corresponding GHI forecasting accuracy. In addition, different LoRa node layouts can be also evaluated in terms of data accuracy and GHI forecasting estimations. In general, higher clear sky GHI ratio values imply more accurate GHI estimations, being less sensitive to loss of data or SF parameter values. Subsequently, low ${\bar{K}}_{t}$ GHI ratio days should be considered for estimating potential errors allowed for short-term forecasting purposes depending on the corresponding loss of data, short time periods, and SF parameters. A sensitive analysis is included in the work from complementary metrics: nRMSE, MAPE, and DTW. These results give additional information to characterize discrepancies among collected and forecast GHI data as a consequence of LoRa parameters and/or node layout. This methodology thus provides a preliminary analysis of potential LoRa network characteristics and sensoring in terms of data accuracy, packets, and GHI forecasting possibilities.

## Figures and Tables

**Table 1:** LoRa/LoRaWAN main characteristics based on the EU 863–870 MHz data rates.

0	SF12/125 kHz	250
1	SF11/125 kHz	440
2	SF10/125 kHz	980
3	SF09/125 kHz	1760
4	SF08/125 kHz	3125
5	SF07/125 kHz	5470
6	SF06/250 kHz	11,000
7	FSK: 50 kbps 440	50,000

**Table 2:** LoRa spreading factor (SF).

Spreading Factor (SF)	Range (Depending on the Terrain)
SF10	8 km
SF09	6 km
SF08	4 km
SF07	4 km

**Table 3:** Time interval between subsequent packets (1% duty-cycle).

**Spreading Factor (SF)**						
SF12	SF11	SF10	SF09	SF08	SF07	SF06
**Time Interval between Subsequent Packets (s)**						
214	115	62	33	18.5	10	6

**Table 4:** Signal to noise ratio (SNR) limits.

Spreading Factor (SF)	Signal to Noise Ratio (SNR) Limit q(j)
SF07	−7.5 dB
SF08	−10.0 dB
SF09	−12.5 dB
SF10	−15.0 dB
SF 11	−17.5 dB
SF 12	−20.0 dB

## Data Availability

The datasets generated and/or analyzed during this study are available from the corresponding author on reasonable request.

## Abbreviations

The following abbreviations are used in this manuscript:

BW	Bandwidth
CSS	Chirp spread spectrum
DTW	Dynamic time warping
GHI	Global horizontal irradiance
ITM	Irregular terrain model
LASSO	Least absolute shrinkage and selection operator
LoRa	Long range
MAE	Mean absolute error
MAPE	Mean absolute percentage error
nRMSE	Normalized root mean square error
PV	Photovoltaic
RF	Random forest
RMSE	Root mean square error
SF	Spreading factor
SNR	Signal to noise ratio
vRES	Variable renewable energy sources
